# Treatment of chronic and complex meniscal tears with arthroscopic meniscus repair augmented with collagen matrix wrapping: failure rate and functional outcomes

**DOI:** 10.1007/s00264-024-06241-1

**Published:** 2024-06-28

**Authors:** Marga H. Vicens, Oriol Pujol, Irene Portas-Torres, Marc Aguilar, Nayana Joshi, Joan Minguell, Enric Castellet, Marcelo Casaccia

**Affiliations:** 1 Departament de Cirurgia I Ciències Morfològiques, Universitat Autònoma de Barcelona, Barcelona, Spain; 2 Reconstructive Surgery of the Locomotor System Group, Vall d’Hebron Research Institute (VHIR), Barcelona, Spain; 3 Knee Surgery Unit, Orthopaedic Surgery Department, Vall d’Hebron University Hospital, Universitat Autónoma de Barcelona, Pg. Vall d’Hebron 119-129, 08035 Barcelona, Spain

**Keywords:** Meniscus, Meniscal tear, Meniscal wrapping, Preservation, Collagen membrane, Arthroscopy

## Abstract

**Purpose:**

Meniscal wrapping is a fully arthroscopic technique that involves enhanced meniscal repair with a tissue-engineered collagen matrix wrapping. This study aims to investigate the feasibility of using the meniscal wrapping technique for the treatment of chronic or complex meniscal tears. The primary objective is to assess its failure rate. The secondary objectives are to analyse complication rate, functional outcomes and overall patient satisfaction.

**Methods:**

This retrospective case series study included patients who sustained chronic and complex tears undergoing meniscal wrapping with autologous liquid bone marrow injection. Failure rate was considered if the patient underwent partial or complete meniscectomy or knee replacement during the follow-up, while other unexpected knee reoperations were considered as complications. Clinical outcomes were evaluated through the IKDC score, Tegner Activity Score and Short Assessment of Patient Satisfaction.

**Results:**

Twenty-one patients were included (15 non-acute bucket-handle tears, three non-acute horizontal tears and three non-acute complex injuries). The failure rate was 9.5% at 33 months. The rate of other unplanned reoperations was 14.3%, but none of these complications were apparently directly related to the wrapping technique. The average postoperative IKDC was 73.3/100. No statistically significant difference was encountered between preinjury and postoperative Tegner Activity Score. The mean overall patient satisfaction was 88.3/100.

**Conclusions:**

Meniscal wrapping can be safely used as an adjunctive technique to meniscal repair in such difficult-to-treat cases to preserve the meniscus. The technique achieves a low failure rate and promising results of knee function, and patient satisfaction.

## Introduction

Meniscal tears are common knee injuries that compromise the meniscus’s ability to convert axial loading into hoop stress, disrupting normal load distribution [1]. When considering its operative treatment, the surgeon should carefully evaluate patient characteristics, knee conditions and tear properties [2]. Traditionally, acute simple peripheral tears in young patients were amenable to repair. On the other hand, chronic or complex tears, specifically those located in the poorly-irrigated white zone, were considered unrepairable owing to their low healing potential, and were managed with meniscectomy. The understanding of the meniscus has evolved, revealing that preserving its anatomy and function is crucial for the knee. Therefore, there has been a shift towards repairing those previously deemed irreparable tears [3]. Several studies show that suture repair of complex tears leads to better functional outcomes and improved quality of life compared to meniscectomy [4–6]. However, it should be noted that failure of sutured complex tears remains an unpredictable concern that warrants attention.

New suturing and augmentation techniques offer promising approaches for the arthroscopic management of complex meniscal tears [7]. One recent augmentation technique to enhance meniscal healing is known as “meniscal wrapping”. It is a fully arthroscopic technique based on a meniscal repair using sutures and enhanced with a collagen matrix wrapping. Furthermore, biologic augmentation, such as autologous liquid bone marrow injection, can be added. Piontek et al. first described this technique in 2012 [8] and, later, they reported two year and five year follow-up clinical studies [9, 10]. They found relatively high survival rate and favourable functional and radiological outcomes, suggesting that combined and complex meniscal tears can be successfully and safely treated with the meniscal wrapping technique. However, these articles were published by the promoters of the technique and limited data exists regarding this procedure.

This study aims to investigate the feasibility of arthroscopic meniscal repair augmented with wrapping technique for the treatment of chronic or complex meniscal tears. The primary objective is to assess its failure rate. The secondary objectives are to analyse complication rate, functional outcomes and overall patient satisfaction. We hypothesize that it may be a preservative option for these difficult-to-treat lesions.

## Materials and methods

### Study design

Following institutional review board (IRB) approval, this study was conducted as a retrospective case series. Consecutive cases of chronic or complex meniscal tears treated with an arthroscopic meniscal repair augmented with a wrapping technique between 2017 and 2023 were identified from our level one university hospital’s institutional database. The authors of this study are not the promoters of the technique [8].

The inclusion criteria were: a) patients who sustained complex and/or non-acute (> 3 months) meniscal tears, b) Use of collagen matrix after suturing to enhance potential healing as an augmentation technique, c) Operated between January 2017 to January 2023 in our institution, d) Age 16–65 years and e) Minimum follow-up of 12 months. The exclusion criteria were: a) Patients with acute meniscal tears (≤ 3 months) that have a simple pattern, b) Injuries treated with simple suturing and c) No post-surgery data.

A meniscal tear was considered chronic if the time elapsed between meniscal injury to surgery was greater than 3 months [11]. On the other hand, we considered complex tears those that so far would have been treated with partial meniscectomy: combined types, white and red-white zones involvement, as well as extensive bucket handle tears [8].

### Outcomes variables

#### Primary endpoint

Meniscal wrapping failure rate. It was considered if the patient underwent partial or complete meniscectomy or knee replacement during the follow-up [9].

#### Secondary endpoints

1. Complication rate: Unexpected knee reoperations (excluding meniscectomy).
2. Functional outcomes: A final outpatient appointment was scheduled and all patients were informed about the study. Patients were interviewed by an investigator of this study to assess the following clinical outcomes: International Knee Documentation Committee—Subjective Knee Evaluation Form (IKDC) and Tegner Activity Score [12].
3. Overall patient satisfaction: It was also assessed during the final outpatient appointment using the Likert scale Short Assessment of Patient Satisfaction Score (SAPS) [13].

#### Other variables

: a) baseline characteristics: age, sex, body mass index (BMI), smoking and pre-injury Tegner score, b) injury characteristics: date, meniscus affected, location, tear type and associated lesions, c) surgery data: date, time to surgery (if it was not possible to determine the exact injury moment or symptoms beginning but they were longer than three months, the tear was considered as chronic), type of meniscal repair and associated injuries management and d) follow-up.

### Surgical technique[8]*:* (Fig. 1)

**Fig. 1:**
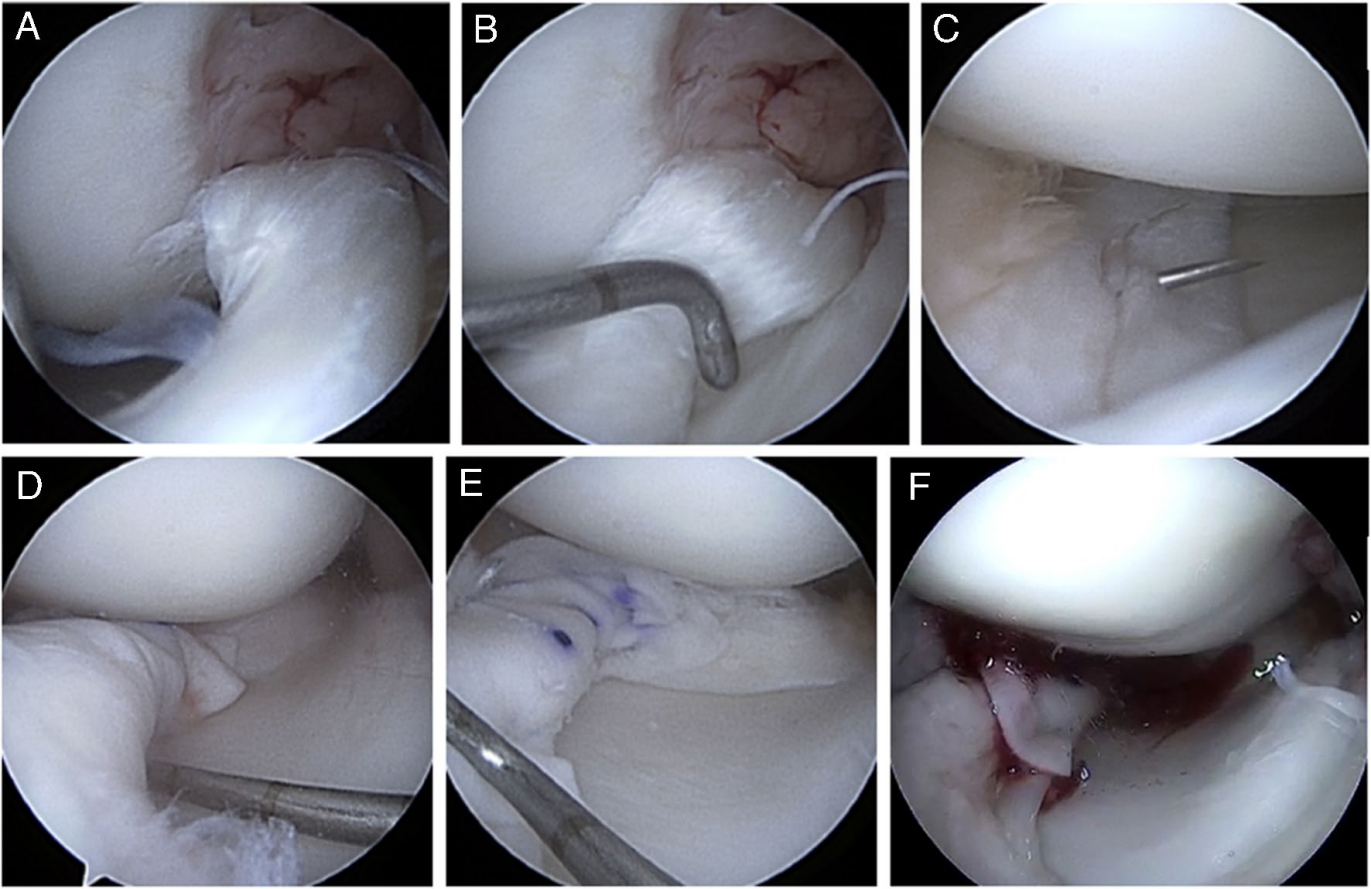
Arthroscopic images showing the surgical technique to perform a meniscal wrapping procedure in a patient with a chronic bucket-handle tear. A The meniscal bucket-handle tear is identified. B The tear is reduced to its anatomical position. C A repair is performed using usual suture techniques. D The collagen matrix is carefully placed to wrap the repaired meniscus. Care should be taken to ensure that the smooth surface is facing the joint space, whereas the porous surface faces the meniscus. E The membrane is fixed around the meniscus using sutures. F Finally, autologous liquid bone marrow (obtained from the intercondylar notch) is injected inside the wrapping

First, a diagnostic knee arthroscopy is made to rule out associated injuries, such as cartilage or ligaments lesions. The meniscal tear is located and the indication to perform the “meniscal wrapping technique” is confirmed (non-acute and complex lesions otherwise scheduled for meniscal removal). Then, the tear is repaired using suture techniques as usual.

The collagen matrix Chondro-Guide (Geistlich Pharma AG, Wolhusen, Switzerland) is introduced inside the joint. It is a bilayer collagen I/III membrane, initially developed for cartilage regeneration, that is biocompatible and naturally resorbed. The smooth top layer is cell-occlusive and prevents cells from diffusing into the joint space. The rough, porous bottom layer adheres to the meniscus keeping the membrane in place and providing a 3D supportive matrix for adhesion and cell differentiation. Using a direct arthroscopic viewing portal, the membrane is carefully placed to wrap the repaired meniscus. It aims to offer a protective environment with a favourable microarchitecture and biological enhancement to optimize meniscus healing. Care should be taken to ensure that the smooth surface is facing the joint space, whereas the porous surface faces the meniscus. The membrane is fixed around the repaired meniscus using suture techniques, improving meniscal wrapping stability and tightening the space between meniscus and collagen matrix to increase the contact surface. Then, autologous liquid bone marrow is obtained from the intercondylar notch and injected inside the wrapping, between the meniscus and the membrane, to provide biological augmentation. Finally, the membrane stability is checked trough flexion–extension knee movement and probe palpation.

### Statistical analysis

Descriptive statistics were used to present the cohort’s characteristics. Categorical variables were described by their absolute value and percentages. The comparison between preinjury and postoperative values of Tegner Activity Score were calculated by nonparametric Wilcoxon test. The cumulative survivorship was studied with the Kaplan–Meier analysis. Continuous variables were presented by their mean, standard deviation, and range. Statistical analysis was conducted using Jamovi 2.4.8 Debug.

## Results

Twenty-one cases fulfilled the inclusion criteria and were included in this study. The 66.7% were males and the mean age was 33.4 years (range: 17–58 years). Mean BMI was 25.4 kg/cm^2^ and 28.6% of patients were smokers. The 85.7% of the lesions affected the medial meniscus. There were 15 (71.4%) non-acute bucket-handle tears, three (14.3%) non-acute horizontal tears and three (14.3%) non-acute complex injuries combining different tear types. Mean time to surgery was 25.2 months. Patient’s baseline characteristics are summarized in Table 1. Five cases (23.8%) presented associated knee lesions: three patients with ACL ruptures (two complete tears and one partial tear with a stable knee) and two with degenerative focal chondral lesions (Table 2).

**Table 1:** Patients baseline characteristics

Case	Age (years)	Sex	BMI (kg/cm^2^)	Smoking	TTS (months)	Meniscus	Tear type
1	50	M	29	Yes	22	Medial	Bucket-handle tear
2	19	M	31	No	24	Medial	Bucket-handle tear
3	38	F	28	No	20	Medial	Bucket-handle tear
4	27	M	26	Yes	21	Medial	Bucket-handle tear
5	34	M	20	Yes	3	Medial	Bucket-handle tear
6	24	M	24	No	58	Medial	Bucket-handle tear
7	27	M	21	No	24	Medial	Bucket-handle tear
8	28	F	26	No	9	Medial	Bucket-handle tear
9	36	F	29	No	10	Medial	Bucket-handle tear
10	22	F	22	No	14	Medial	Bucket-handle tear
11	40	M	25	No	22	Medial	Bucket-handle tear
12	47	F	25	Yes	19	Medial	Bucket-handle tear
13	17	M	21	No	6	Medial	Bucket-handle tear
14	33	M	24	No	26	Lateral	Bucket-handle tear
15	25	M	24	No	13	Medial	Bucket-handle tear
16	32	M	24	No	15	Medial	Complex tear
17	25	M	27	Yes	- (chronic)	Lateral	Horizontal tear
18	45	M	29	No	23	Medial	Complex tear
19	34	F	21	No	33	Medial	Horizontal tear
20	58	F	28	Yes	36	Medial	Horizontal tear
21	41	M	33	No	11	Lateral	Complex tear
Summary (mean [range])	33.4 (17–58)	M: 66.7% F: 33.3%	25.6 (20–33)	28.6%	25.2 (3–58)	Medial: 85.7% Lateral: 14.3%	71.4% Bucket-handle 14.3% Complex 14.3% Horizontal

Legend: *F* female, *M* male, *BMI* Body Mass Index, *TTS* Time To Surgery (time elapsed between injury and surgery)

**Table 2:** Concomitant knee lesions and treatment provided at the time of the index procedure

Case	Meniscal tear type	Concomitant knee injury	Concomitant knee joint treatment
1	Bucket-handle tear	Degenerative focal chondral lesion grade II in the femoral condyle	Debridement
10	Bucket-handle tear	Complete ACL rupture	ACL autologous graft reconstruction
11*	Bucket-handle tear	-Degenerative focal chondral lesion grade II in the patella -Residual knee instability	Lateral extra-articular tenodesis
13	Bucket-handle tear	Partial ACL rupture with stable knee	None
18	Complex tear	Complete ACL rupture	ACL autologous graft reconstruction

Legend: *ACL* anterior cruciate ligament

^*^ Patient with residual rotational instability after previous ACL reconstruction performed 8 years ago

**Fig. 2:**
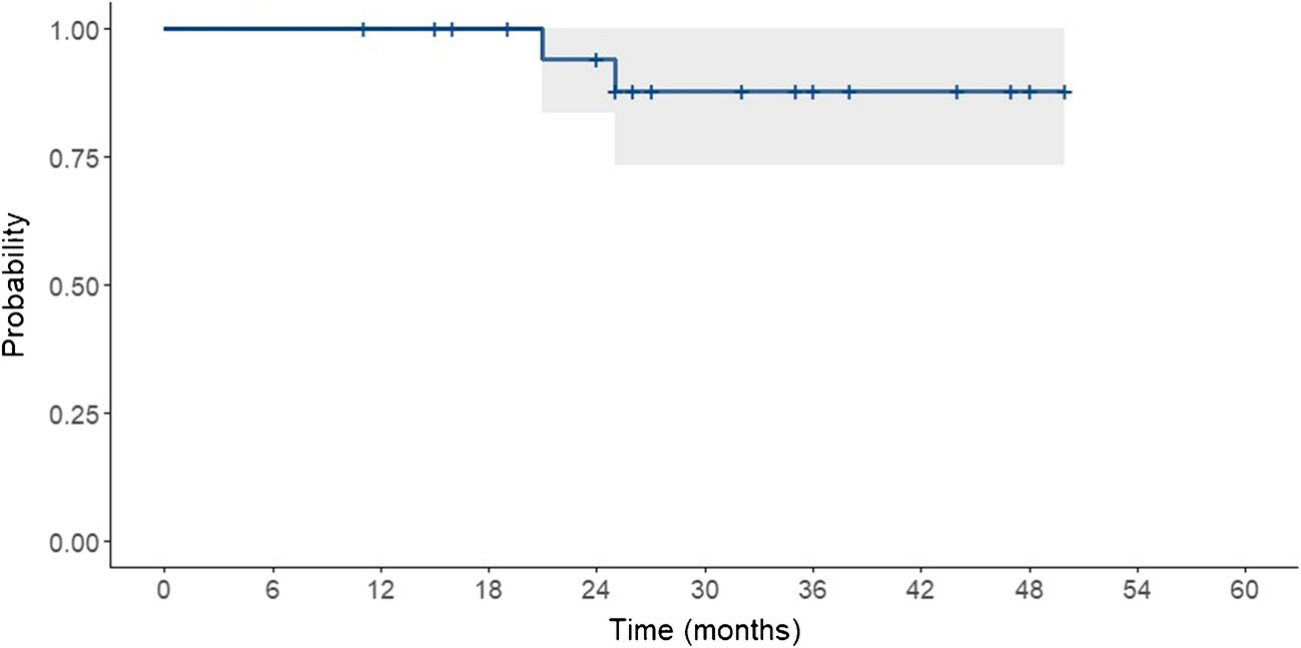
Kaplan–Meier survival curve for meniscal wrapping after complex or chronic tears. Failure was considered if the patient underwent partial or complete meniscectomy or knee replacement (mean follow-up was 33 months)

Two patients required a partial meniscectomy (Fig. 2); therefore, the failure rate was 9.5% after a mean follow-up of 33 months (Table 3). The first patient (case 1) presented persistent knee pain despite achieving satisfactory knee function. An MRI showed a re-tear of the previously repaired meniscus. An arthroscopic partial meniscectomy was performed 21 months after initial treatment. After re-operation, the knee remains pain-free. The second patient (case 8) experienced pain and knee blocking. During a second-look arthroscopy, a meniscal re-tear and an ACL rupture were observed; partial meniscectomy and ACL reconstruction were performed 25 months after surgery. Then, she presented a painless and stable knee.

**Table 3:** Characteristics of cases who required a reoperation

Case	Diagnostic	Symptoms	Reoperation procedure	TTR (months)
Failures				
1	Meniscal re-tear	Persistent knee pain	Partial meniscectomy	21
8	Meniscal re-tear and ACL rupture	Pain and knee blocking	Partial meniscectomy and ACL reconstruction	25
Complications (unplanned reoperation)				
5	Parameniscal cyst	Knee pain	Arthroscopic cyst excision	40
6	Septic arthritis	Fever, knee pain and swelling	Open surgical debridement	26
10	ACL re-rupture	Knee instability	ACL re-reconstruction	14

Legend: *TTR* time to reintervention (time between index surgery and reoperation)

Three patients (14.3%) required unplanned reintervention to the same knee, not apparently directly related to the wrapping technique (Table 3). One patient (case 6) developed septic arthritis after a knee arthrocentesis 26 months after index surgery. He required an open surgical debridement, during which the integrity of the meniscal repair was observed. Another patient (case 5) presented knee pain associated with a parameniscal cyst. A knee arthroscopy was performed to excise the cyst; the meniscus was stable and healed. The remaining patient (case 10) suffered from knee instability after a fall. Subsequent MRI images revealed a complete ACL re-rupture, which was reconstructed afterwards.

Regarding functional outcomes, the mean IKDC score at the end of the follow-up was 73.3 ± 8.08. No statistical differences were encountered between pre-injury and postoperative Tegner Score (2.7 ± 2.05 vs 1.9 ± 0.97, p = 0.114). Finally, the mean patient satisfaction (SAPS) with the procedure was 88.3 ± 9.6/100 points. The 61.1% of them were “very satisfied” and no patient was dissatisfied.

## Discussion

The most important finding of our study is that the failure rate of the meniscal wrapping technique for non-acute and complex meniscal tears is 9.5% after a mean follow-up of 33 months. Furthermore, the rate of other unplanned reoperations is 14.3%. Patients present good to excellent functional outcomes after this augmentation technique, leading to high satisfaction and activity level recovery.

The recognition of the protective role of the meniscus and its importance in the properly knee functioning [1, 2] has led to a shift in treatment philosophy toward a strategy focused on meniscus preservation, as reflected in the ESSKA’s consensus [3, 11]. The self-healing potential of isolated meniscus tears is not clear yet. Only small traumatic tears (< 10 mm) of the lateral meniscus should be left alone and do not require surgery [11]. On the other hand, only 0–35% of patients with degenerative tears treated non-operatively require conversion to surgery [3]. Lee et al. demonstrated superior clinical outcomes with lower likelihood of osteoarthritis development in the repair group compared to meniscectomy [14]. Even in complex tears, some authors have reported better functional outcomes and improved quality of life with meniscal repair. Furthermore, preserving the meniscus leads to greater potential to return to the same level of activity [5, 6].

Nevertheless, suturing complex or chronic tears can be related to a high risk of failure and reoperation. Multiple repairing techniques have been developed to overcome the impaired healing capacity in such difficult-to-treat scenario. Recent advancements in tissue engineering and biological augmentation have expanded the surgical options for meniscal repair [7, 15, 16]. One recent augmentation technique is the meniscal wrapping, based on meniscal repair enhanced with collagen matrix wrapping [8–10].

In the authors opinion, acute meniscal tears are usually amenable to simple repair techniques, which should be the first surgical choice. However, the meniscal wrapping procedure offers an additional tool to save the meniscus in patients with non-acute and complex lesions otherwise scheduled for meniscal removal. This technique was first described by Piontek et al. [8]. It uses a bilayer collagen I/III membrane that combines a cell-occlusive top layer that prevents progenitor cells from diffusing into the articular space and a rough, porous bottom layer that adheres to the meniscus and provides a 3D supportive matrix for adhesion and cell differentiation. The wrapping offers a protective environment with a favourable microarchitecture and biological enhancement to optimize meniscus healing. In a biomolecular study, it was found that the membrane plays a role in facilitating cell adhesion, proliferation and the release of cytokines related to tissue regeneration and remodelling [17]. In a rabbit model, Nakagawa et al. demonstrated that the wrapping treatment induced fibrochondrocyte-like cells and collagen, providing better meniscal regeneration that the simple repair group [18]. Moreover, during the procedure, we regularly inject autologous liquid bone marrow inside the wrapping to provide additional biologic support. It can be obtained in a relatively simple and inexpensive method, and it is a known source of stem cells and growth factors. It may provide a similar effect as the obtained through the ACL reconstruction tunnels, which have demonstrated to reduce the risk of failure of the meniscal repair [19]. Other authors also defend that further biological augmentation can be achieved by using bone marrow [10, 17, 18, 20]. Bakowski et al. proved a good survival and viability of the bone marrow-derived cells seeded on the membrane, suggesting its beneficial effect on the healing process [17]. In a preliminary clinical study, Whitehouse et al. analysed a therapy combining undifferentiated mesenchymal stem cells with a collagen scaffold to drive healing of avascular meniscal tears, suggesting that its repair is possible through this biological enhanced procedure [20].

The present study demonstrates a failure rate of 9.5% at mid-term follow-up when treating non-acute and complex meniscal tears using the wrapping technique. Only two cases (2/21) required partial meniscectomy as salvage procedure due to recurrent meniscal tears (31 and 25 months after surgery, respectively). In these two cases, the amount of resected meniscus seemed to be less than it would have been if the meniscectomy had been initially performed. However, these cases showed the worst IKDC results at final follow-up. Piontek et al. demonstrated a similar failure rate (12% at 5 years) in a case series of 44 patients with complex meniscus tears and lesions located in the avascular zone treated with the meniscal wrapping technique [9, 10]. Furthermore, they defended that the procedure leads to a long-term stable meniscus preservation with a significant improvement of clinical and MRI-based outcomes. As far as we are concerned, these are the only previous studies that has investigated meniscal wrapping survival rate at mid-term follow-up.

Our outcomes compare favourably to the failure rates reported when these challenging lesions are treated using non-augmented repair techniques [21–23]. A recent metanalysis, that included 27 studies with a minimum follow-up of five years, demonstrated an overall failure rate of 22.6%, and a pooled modern devices failure rate of 19.5% [24]. They stated that early-generation devices had significantly higher failure rates than modern techniques. These results are comparable to those published in another metanalysis by Schweizer et al. [25]; their overall failure rate after meniscal repair was 19.1% at 7.1 years. Nonetheless, new augmentation techniques, were not included in both studies.

In our series, the mean IKDC score at final follow-up was 73.3. Furthermore, patients recovered their pre-injury activity level and presented high satisfaction with the procedure. Our functional results are comparable to other studies. A postoperative IKDC of 74.8 was encountered in a sample of bucket-handle tears which underwent meniscal repair [26]. Piontek et al. also found that the wrapping technique provided very good mid-term clinical outcomes with significant improvement in subjective scores five years after surgery [9, 10]. We defend that clinical outcomes of the meniscal wrapping are encouraging, considering the complexity of their target meniscal tears.

The wrapping procedure has demonstrated to be safe. In our study, only three cases required unplanned reoperation (14.3%). It is important to highlight that none of these complications were apparently directly related to the wrapping technique. One patient developed a septic arthritis after an arthrocentesis (26 months after surgery), another one suffered from ACL re-rupture after a fall and the last one presented knee pain associated with a parameniscal cyst. An arthroscopy was performed 39 months after the index procedure; it could be observed that the meniscal tear was healed and stable and that the cyst was seated over the trimmed end of the meniscal suture. Therefore, the cyst was excised and suture was removed.

We recognize the limitations of the present research. First, it is a retrospective study without a control group. We don’t compare the outcomes obtained through the meniscal wrapping technique with other techniques. Second, the preoperative IKDC score was not collected. Therefore, we cannot analyse its modification with the surgical procedure. Third, nor radiographic nor second-look arthroscopic examinations were conducted to prove the effectiveness of the technique to repair the meniscus. However, clinical success was considered if the patients required no meniscectomy during the follow-up. On the other hand, this research has also some strengths. It is one of the largest and more complete study analysing the role of this promising meniscal preserving technique. We have not only assessed its failure rate but also patients satisfaction and functional outcomes. The authors of this study are not the promoters of the technique.

## Conclusions

The arthroscopic meniscal repair augmented with the wrapping technique can be an effective and safe preservative treatment to manage chronic or complex meniscal tears. It has showed a 9.5% failure rate after a mean follow-up of 33 months. Patients presented good to excellent functional outcomes, leading to high satisfaction and activity level recovery. No unplanned reoperation directly attributable to the wrapping procedure itself was observed in this study.

## Data Availability

Data available on request from the authors.
